# Impact of serum neurofilament light on clinical decisions in a tertiary multiple sclerosis clinic

**DOI:** 10.1177/13524585241277044

**Published:** 2024-10-17

**Authors:** Zoë YGJ van Lierop, Mark HJ Wessels, Womei ML Lekranty, Bastiaan Moraal, Sam N Hof, Laura Hogenboom, Brigit A de Jong, Nandi Meijs, Liselore A Mensing, Bob W van Oosten, Nik Sol, Zoé LE van Kempen, Lisa Vermunt, Myrthe J Willems, Eva MM Strijbis, Bernard MJ Uitdehaag, Joep Killestein, Charlotte E Teunissen

**Affiliations:** Department of Neurology, Amsterdam UMC (Location VUmc), Vrije Universiteit Amsterdam, MS Center Amsterdam, Amsterdam Neuroscience, Amsterdam, The Netherlands; Department of Neurology, Amsterdam UMC (Location VUmc), Vrije Universiteit Amsterdam, MS Center Amsterdam, Amsterdam Neuroscience, Amsterdam, The Netherlands; Department of Neurology, Amsterdam UMC (Location VUmc), Vrije Universiteit Amsterdam, MS Center Amsterdam, Amsterdam Neuroscience, Amsterdam, The Netherlands; Department of Radiology and Nuclear Medicine, Amsterdam UMC, Vrije Universiteit Amsterdam, MS Center Amsterdam, Amsterdam Neuroscience, Amsterdam, The Netherlands; Department of Neurology, Amsterdam UMC (Location VUmc), Vrije Universiteit Amsterdam, MS Center Amsterdam, Amsterdam Neuroscience, Amsterdam, The Netherlands; Department of Neurology, Amsterdam UMC (Location VUmc), Vrije Universiteit Amsterdam, MS Center Amsterdam, Amsterdam Neuroscience, Amsterdam, The Netherlands; Department of Neurology, Amsterdam UMC (Location VUmc), Vrije Universiteit Amsterdam, MS Center Amsterdam, Amsterdam Neuroscience, Amsterdam, The Netherlands; Department of Neurology, Zuyderland Hospital, Sittard-Geleen, The Netherlands; Department of Neurology, Spaarne Gasthuis, Haarlem, The Netherlands; Department of Neurology, Amsterdam UMC (Location VUmc), Vrije Universiteit Amsterdam, MS Center Amsterdam, Amsterdam Neuroscience, Amsterdam, The Netherlands; Department of Neuro-Oncology, Antoni van Leeuwenhoek Hospital, Amsterdam, The Netherlands; Department of Neurology, Amsterdam UMC (Location VUmc), Vrije Universiteit Amsterdam, MS Center Amsterdam, Amsterdam Neuroscience, Amsterdam, The Netherlands; Neurochemistry Laboratory, Department of Clinical Chemistry, Amsterdam UMC, Vrije Universiteit Amsterdam, Amsterdam Neuroscience, Amsterdam, The Netherlands; Department of Neurology, Diakonessenhuis, Utrecht, The Netherlands; Department of Neurology, Amsterdam UMC (Location VUmc), Vrije Universiteit Amsterdam, MS Center Amsterdam, Amsterdam Neuroscience, Amsterdam, The Netherlands; Department of Neurology, Amsterdam UMC (Location VUmc), Vrije Universiteit Amsterdam, MS Center Amsterdam, Amsterdam Neuroscience, Amsterdam, The Netherlands; Department of Neurology, Amsterdam UMC (Location VUmc), Vrije Universiteit Amsterdam, MS Center Amsterdam, Amsterdam Neuroscience, Amsterdam, The Netherlands; Neurochemistry Laboratory, Department of Clinical Chemistry, Amsterdam UMC, Vrije Universiteit Amsterdam, Amsterdam Neuroscience, Amsterdam, The Netherlands

**Keywords:** Multiple sclerosis, biomarkers

## Abstract

**Background and objectives:**

Serum neurofilament light (sNfL) is a biomarker for neuro-axonal damage in multiple sclerosis (MS). Clinical implementation remains limited. We investigated the impact of implementation on clinical decisions using questionnaires at the MS Center Amsterdam, a tertiary outpatient clinic.

**Methods:**

sNfL assessments were added to routine clinical practice (August 2021–December 2022). Before and after the results, clinicians filled in questionnaires on context of testing, clinical decisions, certainty herein, expectation of magnetic resonance imaging (MRI) activity, urgency, and motivation to receive the sNfL result and perceived value of sNfL.

**Results:**

sNfL was assessed in 166 cases (age 41 ± 12 years, 68% female, 64% disease-modifying therapy (DMT) use) for the following contexts: “DMT monitoring” (55%), “new symptoms” (18%), “differential diagnosis” (17%), and “DMT baseline” (11%). Clinical decisions changed in 19.3% of cases post-disclosure, particularly in context “new symptoms” (38%) and with higher sNfL levels (β = 0.03, *p* = 0.04). Certainty increased (*p* = 0.004), while expectation of MRI activity decreased with disclosure of low sNfL levels (*p* = 0.01). Motivation was highest in context “differential diagnosis” (*p* < 0.001); perceived value and urgency were highest in context “new symptoms” (*p* = 0.02).

**Conclusion:**

In this study, sNfL implementation had considerable impact on clinical decision-making and certainty herein. Standard implementation may complement patient care but warrants caution and more exploration in diverse clinical settings

## Introduction

Multiple sclerosis (MS) affects the central nervous system (CNS) and is defined by acute occurrences of neurological deficits caused by inflammatory demyelination (relapses), and disability accumulation caused by neurodegenerative mechanisms (progression).^1^ Although numerous increasingly effective disease-modifying treatments (DMTs) have become available, disease activity (as determined by relapse rate and focal magnetic resonance imaging (MRI) lesions) still occurs in a considerable portion of patients.^2,3^ It is therefore important to be able to prognose these patients that may potentially experience disease activity and monitor their therapeutic response. Proposed clinical and MRI parameters have been able to do so on a group level, but biomarkers able to perform this on individuals are needed.^4,5^ Molecular biomarkers could potentially serve this purpose, as they allow quantifiable and standardized implementation.^5,6^ A blood-based biomarker could outdo cerebrospinal fluid (CSF)-based biomarkers in practice since blood drawing is minimally invasive and often applied in MS outpatient clinics in the context of differential diagnosis, excluding infections in possible (pseudo) relapses and DMT monitoring.^7,8^

Serum neurofilament light (sNfL) is the most extensively studied and promising MS biomarker particularly in the context of disease activity. It has been shown to reflect acute disease activity (both clinical and radiological), disease and disability progression, and treatment response.^9–11^ Furthermore, robust and accurate quantification techniques,^12^ standard operating procedures,^13^ and online applications have been developed to aid the interpretation of an individual sNfL value.^14^ Together, these advancements have paved the way toward the implementation of sNfL in clinical practice.^15,16^

However, current knowledge about the added value of sNfL implementation for clinical decision-making is limited, causing a gap between the use of sNfL for MS research and clinical practice. The design of practical guidelines for clinicians could at least partly bridge this gap.^8,17^ Therefore, in this study, we aimed to gain knowledge about the impact of sNfL implementation on clinical decision-making in a tertiary MS clinic. We aimed to study in what context physicians might use sNfL, what the effect of measuring sNfL in each context might be, whether testing sNfL was perceived as valuable, and finally assess in what scenarios implantation might indeed be fruitful and useful. Using a two-part questionnaire filled out by clinicians before and after sNfL assessment, our primary objective was to assess whether sNfL altered clinical decision-making (such as preferring additional brain MRI or switching DMT), level of certainty herein, and expectation of MS activity on MRI. Secondary objectives were to investigate the association of clinical characteristics with aforementioned changes, the level of urgency and motivation to receive the sNfL result, and ultimately, the perceived overall value of sNfL in different clinical contexts.

## Methods

### Questionnaires

During the study period from August 2021 to December 2022, sNfL was included in the routine practice of laboratory assessments at the outpatient clinic of the MS Center Amsterdam. Following each routine sNfL assessment during this period, clinicians automatically received a link to the study questionnaire (Supplemental material 1). It was explained that filling out the questionnaire was voluntary, and clinicians could also opt out. The questionnaire was subdivided into Part 1, filled out before disclosure of the sNfL result, and Part 2, filled out after disclosure of the sNfL result. Provided that Part 1 of the questionnaire was completed and the sNfL result was disclosed, clinicians automatically received a second link to Part 2 of the questionnaire. In Part 1 (Question 1), clinicians could choose one of the four contexts to measure sNfL: (1) “differential diagnosis” (MS vs other diagnosis), (2) “new symptoms” in MS, (3) “DMT monitoring,” and (4) “DMT baseline.” Next, clinical decision-making was investigated through context-specific multiple-choice options. The following questionnaire items were designed as a five-point Likert-type scale: the level of certainty about prior clinical decision-making, expectation of MS activity on follow-up brain MRI (Part 1 and Part 2), and the level of urgency and motivation to measure sNfL (Part 1). In the final questionnaire item (Part 2), clinicians ranked their overall perceived value of sNfL in the context of the case on a scale of 1 (*lowest value*) to 10 (*highest value*). The “differential diagnosis” answers are shown in Supplemental material 2.

### Case selection

A case was defined as: “a consultation between a patient and a clinician that included a sNfL assessment.” Cases that complied with the following criteria were included in the data analysis: (1) patient written informed consent for participation in the ongoing observational Amsterdam MS cohort study,^18^ (2) disclosure of the sNfL result after consultation, (3) completion of Part 1 of the questionnaire before disclosure of the sNfL result by the clinician, and (4) completion of Part 2 of the questionnaire by the clinician. Clinical data were collected using the Amsterdam MS cohort study database (Table 1) and included sex, age, diagnosis, MS subtype, any DMT use, first- or second-line DMT use, months of DMT use, the sNfL level (pg/mL), months to last relapse, any MS activity on the last brain MRI, and clinicians’ clinical experience. The date of the blood draw used for sNfL analysis was considered the baseline timepoint for clinical data.

**Table 1.** Baseline characteristics for the 166 included cases.

Baseline characteristics^a^	Total (*n* = 166)^b^	DMT efficacy (*n* = 82, 49%)	New MS symptoms (*n* = 29, 18%)	Differential diagnosis (*n* = 28, 17%)		Screening DMT (*n* = 27, 16%)
				CIS or MS (*n* = 20)	No MS (*n* = 8)	
Females (*n*, %)	112 (68)	56 (68)	20 (69)	11 (55)	5 (63)	20 (74)
Age at sNfL blood sample (years ± SD)	41 ± 12	42 ± 11	39 ± 13	44 ± 13	41 ± 15	37 ± 11
Subtypes (*n*, %)						
CIS	3 (2)	0	0	2 (10)		1 (4)
RRMS	132 (80)	73 (89)	24 (83)	12 (60)		23 (85)
SPMS	10 (6)	5 (6)	4 (14)	1 (5)		0
PPMS	13 (8)	4 (5)	1 (3)	5 (25)		3 (11)
Disease-modifying therapies (%)						
None	60 (36)	4 (5)	8 (28)	16 (85)	8 (100)	23 (85)
Ocrelizumab	44 (27)	32 (39)	10 (35)	1 (5)		1 (4)
Dimethyl fumarate	26 (16)	17 (21)	6 (21)	1 (5)		2 (7)
Teriflunomide	9 (5)	8 (10)	1 (3)	0		0
Interferon-beta	9 (5)	6 (7)	1 (3)	1 (5)		1 (4)
Fingolimod	5 (3)	4 (5)	1 (3)	0		0
Natalizumab	4 (2)	3 (4)	1 (3)	0		0
Glatiramer acetate	3 (2)	3 (4)	0	0		0
Cladribine	3 (2)	3 (4)	0	0		0
Ozanimod	2 (1)	1 (1)	1 (3)	0		0
Rituximab	1 (1)	1 (1)	0	0		0
MRI activity prior to sNfL (0.53 ± 1.4 months)^c^	63 (39)	30 (37)	13 (45)	7 (35)	-	13 (48)
MRI activity after sNfL (3.75 ± 2.3)^c^	26/96 (27)	9/44 (20)	5/23 (22)	5/8 (63)	-	6/22 (27)
sNfL—mean ± SD (pg/mL)	13 + 17	10 *±* 5.8	15 *±* 18	25 *±* 39	16 *±* 14	11 *±* 8
sNfL—age-corrected percentiles (%)^d^						
<5	1 (1)	0	0	1 (5)	0	0
5–10	4 (2)	3 (4)	1 (3)	0	0	0
10–25	13 (8)	9 (11)	3 (10)	0	0	1 (4)
25–50	42 (25)	24 (29)	6 (21)	3 (15)	2 (25)	7 (26)
50–75	60 (36)	29 (35)	12 (41)	6 (30)	2 (25)	11 (41)
75–90	15 (9)	5 (6)	2 (7)	2 (10)	0	6 (22)
90–95	8 (5)	3 (4)	1 (3)	3 (15)	1 (13)	0
>95	23 (14)	9 (11)	4 (14)	5 (25)	3 (38)	2 (7)

DMT: disease-modifying therapies; MS: multiple sclerosis; CIS: clinically isolated syndrome; SD: standard deviation; RRMS: relapsing–remitting multiple sclerosis; SPMS: secondary progressive multiple sclerosis; PPMS: primary progressive multiple sclerosis; MRI: magnetic resonance imaging; sNfL: serum neurofilament light.

a The date of the blood draw for sNfL measurement was defined as baseline time point.

b This included 157 unique patients, for sNfL was measured in three separate consultations by one patient, and two separate consultations in seven patients.

c MRI activity (new/enlarged T2 lesions and T1 gadolinium-enhanced (T1GE) lesions) prior to the sNfL result was determined in 158 scans of patients with MS. MRI activity after the sNfL result was determined in 96 scans of patients with MS.

d Calculated using the NfL interface for physicians. For patients with CIS or MS, we calculated age-corrected percentiles based on a reference cohort of patients with MS. For some age groups of patients with MS (below 20 and above 62 years of age), age-corrected percentiles are technically unknown because of insufficient data. In this study, we therefore extrapolated age-corrected percentiles based on the available data for MS patients. For patients without MS, we calculated age-corrected percentiles based on a reference cohort of controls (i.e. without a neurological diagnosis).

### Clinicians

A total of 20 clinicians completed the questionnaires. Among them were five neurologists, nine neurology residents, and four physician researchers with a specialty in MS. At the start of this study, all clinicians in the MS center received a link to an interactive manual (https://mybiomarkers.shinyapps.io/Neurofilament/) to aid their interpretation of the sNfL result.^19^ In the interactive manual, sNfL is corrected for age.^14^

### Serum NfL measurement

Serum samples were collected at the outpatient laboratory of Amsterdam University Medical Center (UMC), location VU medical center. Centrifugation (1800 g, 10 min at room temperature) was performed within 2 hours and serum samples were stored at −20°C. sNfL analysis was scheduled weekly on Thursdays, and results were reported to clinicians the next day (including the link to the interactive manual). After collection from the freezer, samples were thawed and prepared according to standard operating procedures for the Simoa NF-light^®^ Advantage Kit (Quanterix, Billerica, USA) as described before.^20^

### Magnetic resonance imaging

To investigate the influence of radiological activity on clinicians’ answers, we collected data from brain MRI at two time points. First, the most recent MRI prior to the sNfL sample date (baseline), and second, the first available MRI after baseline, which was performed in 105 (63%) of 166 cases within the study period. The mean time interval between the most recent MRI prior to the sNfL sample date was 0.53 ± 1.4 months. The mean time interval between the sNfL sample date and the first available MRI after baseline was 3.75 ± 2.3 months. Radiological disease activity was defined as new/enlarged T2 hyperintense lesions and T1 gadolinium-enhanced (T1GE) lesions reported by neuroradiologists blinded to the sNfL results.

### Data analyses

We used the related-samples Wilcoxon Signed-Rank Test to study the change in (Likert-type scale-based scores of) certainty about clinical decision-making and expectation of MS activity on follow-up brain MRI between Part 1 (before disclosure of the sNfL result) and Part 2 of the questionnaire (after disclosure of the sNfL result). We compared the proportions of changed clinical decisions, certainty herein, and expectation of MS activity on MRI between the clinical contexts “differential diagnosis,” “new symptoms” in MS, and “DMT monitoring” using a chi-square test. Since clinical decisions were already made in the context of “DMT baseline,” it was excluded from these analyses (and questionnaire items). sNfL levels were compared between “high,” “neutral,” and “low” expectation of MRI activity by independent-samples Kruskal–Wallis test. Clinical characteristics associated with either a change in clinical decisions or certainty herein were identified by logistic regression analysis. These included any DMT use, first- or second-line DMT use, months of DMT use, the sNfL level (pg/mL), months to last relapse, and any MS activity on the last brain MRI. Furthermore, we investigated the association between a change in clinical decisions and questionnaire-based data, regarding certainty about clinical decisions before and after disclosure of the sNfL result and the motivation to receive the sNfL result and the perceived value of the sNfL result after disclosure. To compare the level of urgency and motivation to receive the sNfL result, and the perceived value of the sNfL result between all four clinical contexts, we applied an independent-samples Kruskal–Wallis test. Factors associated with a higher perceived value of the sNfL result were identified by linear regression analysis, for which all previously described clinical characteristics were included. A *p*-value of less than 0.05 was considered statistically significant. Statistical analyses were performed with IBM SPSS Statistics Version 26.0 (Armonk, NY: IBM Corp) and R statistical software version 4.0.3.

### Standard protocol approvals, registrations, and patient consents

The Institutional Review Board (Medical and Biobank ethics Committee of Amsterdam UMC, location VUmc) approved the use of routine medical files for research purposes (registration no. 2016.554). All patients gave written informed consent for the collection and use of medical data and biological fluids for research purposes. This study adhered to the ethical principles of the Declaration of Helsinki.

## Results

### Baseline characteristics

Part 1 of the survey was filled out for a total of 173 cases. The sNfL result was missing in 15 cases, supposedly because patients did not visit the laboratory to draw the blood sample. Part 2 of the questionnaire was completed in all remaining cases, leading to a total number of 166 complete cases. This included 157 unique patients, for sNfL was measured in three separate consultations by one patient, and two separate consultations in seven patients.

The mean age of the 166 complete cases was 41 ± 12 years and 68% was female (Table 1). The most common clinical context where NfL was requested was “DMT monitoring” (49%), followed by “new symptoms” in patients with MS (18%), “differential diagnosis” (17%), and finally “DMT baseline” (16%). The majority of cases (64%) were treated with DMT at baseline, mostly ocrelizumab (28%) or dimethyl fumarate (16%). MRI activity prior to the sNfL result was found in 39% of all cases, mostly in the context of new MS symptoms, and after the sNfL result in 27% of cases, mostly in the context of differential diagnosis.

Except for one case, all “differential diagnosis” cases were second opinions for clinicians outside of our center. In 12 (42%) of these cases, clinicians did not report any differential diagnosis besides MS, before nor after sNfL disclosure. Instead, clinicians indicated that the objective of the second opinion in these cases was to differentiate between MS subtypes or advice on DMT choice (Supplemental material 2).

### Change in clinical decisions after sNfL disclosure

**Figure 1.**
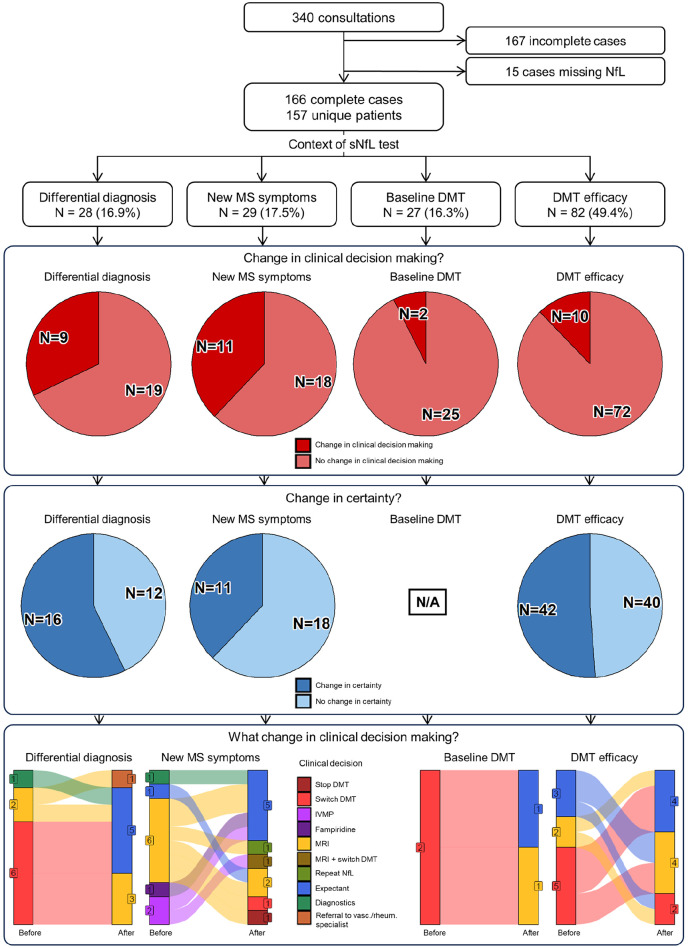
Flowchart with pie charts and alluvial plots depicting inclusions, divisions into the contexts of use, change in certainty, and changes in clinical decision-making after sNfL disclosure. sNfL: serum neurofilament light chain; DMT: disease-modifying therapy; MS: multiple sclerosis; N/A: not applicable.

In 32 (19.3%) of all cases, clinical decisions changed after disclosure of the sNfL result (Figure 1). This percentage was significantly higher in the context of “new symptoms” (38%, *p* = 0.002) than in the contexts of “differential diagnosis” (32%), “DMT monitoring” (12%), and “DMT baseline” (6%).

A higher sNfL level was associated with a change in clinical decisions after disclosure of the sNfL result (β = 0.03, *p* = 0.04). The other clinical characteristics such as DMT use, months to last relapse, and MS activity on MRI showed no significant association. Questionnaire-based variables significantly associated to a change in clinical decisions included a lower baseline certainty about clinical decisions (β = −0.71, *p* = 0.01), a higher motivation to receive the sNfL result (β = 0.52, *p* = 0.01), and a higher perceived value of the sNfL result (β = 0.33, *p* = 0.01).

### Certainty about clinical decisions

**Figure 2.**
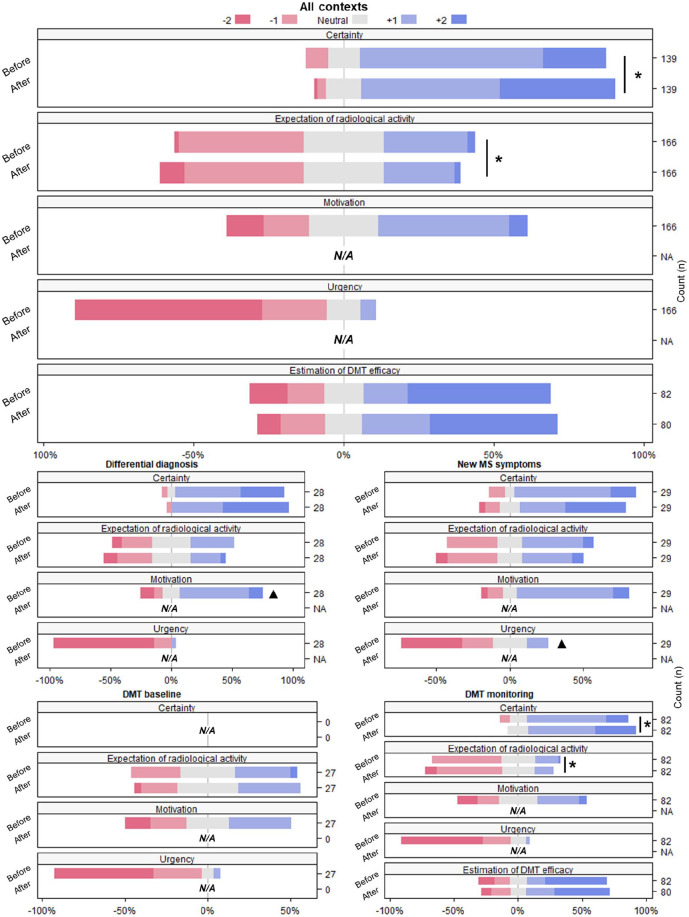
Likert-type scales showing questionnaire response for all contexts combined and different context separately. The median scores of questionnaire items were compared before and after sNfL disclosure, and significant differences between timepoints (*p* < 0.05) are indicated with *. The median scores were furthermore compared between contexts, and significant differences between contexts (*p* < 0.05) are indicated with ^▲^. N/A: not applicable/not asked; sNfL: serum neurofilament light; DMT: disease-modifying therapy; MS: multiple sclerosis.

Excluding the context “DMT baseline,” certainty about clinical decisions was analyzed in 139 cases. In 50% of these cases, certainty changed after disclosure of the sNfL result (Figure 1). As shown in Figure 2, certainty was already moderate (61%) to high (21%) before disclosure of the sNfL result. Overall, certainty significantly increased after disclosure of the sNfL result (*p* = 0.004). In all clinical contexts together, 17% of cases showed decreased, 50% equal, and 34% increased certainty about clinical decisions. The proportion of changed certainty did not significantly differ among the clinical contexts of “differential diagnosis” (43%), “DMT monitoring” (49%), and “new symptoms” (62%). Furthermore, the proportion of either increased, equal, or decreased certainty did not significantly differ among the clinical contexts.

A higher motivation to receive the sNfL result was the only questionnaire-based variable with significant association to a change in certainty (β = 0.33, *p* = 0.03). The perceived value of the sNfL result and the clinical characteristics such as DMT use, months to last relapse, MS activity on MRI, and the sNfL level showed no significant association.

### Urgency and motivation to receive the sNfL result

The majority of clinicians indicated an urgency to receive the sNfL result, namely 55% of cases within 7 days, 33% within 3–4 days, 14% within 1 day, and 5% within 2 hours. Clinicians were never neutral with respect to urgency. Comparing between the different clinical contexts, “new symptoms” showed a significantly higher urgency (14% within 2 hours, 24% within 1 day) compared to the other contexts (*p* = 0.02, Figure 2).

The motivation to receive the sNfL result was high in 12% of all cases, moderate in 42% of cases, neutral in 23% of cases, moderately low in 14% of cases, and low in 10% of cases.

In the context of “differential diagnosis,” the level of motivation to measure sNfL was significantly higher (57% moderate, 11% high) compared to other contexts (*p* ⩽ 0.001, Figure 2).

### Expectation of MS activity on follow-up brain MRI

Expectation of MRI activity was “unlikely” in the larger portion of cases (42%) before disclosure of the sNfL result (Figure 2). The sNfL level was significantly higher in the group with high expectation of MRI activity before (*n* = 50, median 10 pg/mL (8–19), *p* = 0.03) and after disclosure of the sNfL result (*n* = 42, median 12 pg/mL (9–29), *p* < 0.001). Overall, the expectation of MRI activity significantly decreased after the disclosure of the sNfL result, (*p* = 0.008). The level of expectation decreased in 27% of cases (median sNfL 8 pg/mL (6–10)) remained equal in 58% of cases (median sNfL 9 pg/mL (6–13)), and increased in 15% of cases (median sNfL 15 pg/mL (8–27)). This was in accordance with a significantly higher median sNfL level in the group with increased expectation (*p* = 0.002). The proportion of either increased, equal, or decreased expectation did not significantly differ among clinical contexts. In nine cases, clinicians decided to perform an MRI scan after the disclosure of the sNfL result (median 22 pg/mL (10–60)) instead of other follow-up actions.

### Estimated DMT efficacy in the context of “DMT monitoring”

In the context of “DMT monitoring” (*n* = 82), estimated DMT efficacy was already high for most cases (48%) before the disclosure of the sNfL result (Figure 2). Estimated DMT efficacy did not significantly change after the disclosure of the sNfL result. Estimated DMT efficacy decreased in 20% of cases (median sNfL 10 pg/mL (6-15)), remained equal in 60% of cases (median sNfL 8 pg/mL (6-10)), and increased in 21% of cases (median sNfL 9 pg/mL (5–12)). Median sNfL levels did not significantly differ among the groups of decreased, equal, and increased estimated DMT efficacy.

### Perceived value of the sNfL result

On a scale of 1–10, clinicians’ median perceived value of the sNfL result was 6 (5–7). This score was significantly higher in the context of “new symptoms” (7 (6–8) *p* = 0.005) compared to “differential diagnosis” (6 (5–8)), “DMT monitoring” (6 (4–7)), and “DMT baseline” (5.5 (4.75–6.25)).

We next investigated which factors were associated with a higher perceived value of the sNfL result. We found that a change in certainty (not the absolute levels before or after disclosure of the sNfL result) about clinical decisions (stand. β = 0.21, *p* = 0.01), a higher motivation to receive the sNfL result (stand. β = 0.36, *p* < 0.001), and fewer months since the last relapse (stand. β = −0.18, *p* = 0.03) were associated with a higher perceived value of the sNfL result. Furthermore, the perceived value of the sNfL result was higher in cases without DMT use compared to any DMT use (*p* = 0.03), and in cases with first-line DMT use compared to second-line DMT use (*p* = 0.04). The remaining clinical characteristics such as MS activity on MRI and the sNfL level showed no significant association with the perceived value of NfL.

In 52 cases, a previous sNfL result was available with a median value of 8.6 pg/mL (6.7–12.9). Compared to the previous sNfL result, in 17 cases there was a slight increase (ranging from 0.1 to 4.9) and in the remaining 35 cases a decrease (ranging from −30.6 to −0.1) in sNfL. This has influenced clinicians’ answers to the questionnaire, as we found that the estimation of DMT efficacy (DMT monitoring group) changed in a significantly higher proportion of cases compared to the cases without a previous sNfL value (*p* = 0.017). The proportion of cases with a change in clinical decisions, a change in certainty about clinical decisions, and changed expectation of MRI activity did not significantly differ between cases with and without a previous sNfL value, nor did the level of urgency and motivation to measure sNfL and the perceived value of sNfL. Whether sNfL decreased or increased when compared to an available previous measurement, had no significant effect on certainty about clinical decision-making.

## Discussion

This study investigated the impact of sNfL implementation into routine practice at a tertiary MS clinic, which to our knowledge has not been reported before. We compared clinicians’ questionnaire-based clinical decisions, certainty herein and expectation of MS activity on follow-up brain MRI before (Part 1) and after (Part 2) a total of 166 sNfL assessments in the contexts of “DMT monitoring,” “new symptoms” in MS, “differential diagnosis” (MS vs other diagnosis), and “DMT baseline.” We found that a change in clinical decisions after disclosure of the sNfL result occurred in almost 20% of all cases, mostly in the context of “new symptoms” and “differential diagnosis.” In the context of “new symptoms” and “differential diagnosis,” the proportion of change in clinical decisions was relatively higher compared to the contexts “DMT monitoring” and “DMT baseline,” in accordance with a significantly higher perceived value of the sNfL result (new symptoms), and higher urgency (new symptoms) and motivation (differential diagnosis) to receive the sNfL result. Furthermore, we found a significant increase in clinicians’ certainty about clinical decisions after disclosure of the sNfL result, and a significant decrease in their expectation of MS activity on follow-up brain MRI.

Strengths of this study include the novelty of investigating the impact of sNfL on daily practice in a real-world situation. Furthermore, our results inquire about clinicians’ perceived value of standard sNfL implementation, and by use of a published web-based tool to aid their interpretation of the sNfL result. We did not find any previous scientific studies to compare with our results. We acknowledge several possible limitations of this study. Since this is a single-center study in a tertiary clinic where the MS population is relatively complex and mostly treated with highly effective DMT such as ocrelizumab, our results could be less representative for MS clinics with different population characteristics. We emphasize the relatively higher proportion of changed clinical decisions and motivation in the context of “differential diagnosis” should be interpreted with caution. sNfL was used in these cases to differentiate between MS subtypes, or between MS and other neurological pathologies. We note that our results within this scenario probably reflect clinicians’ preference for specific differential biomarkers as tools to support pathology differentiation in daily practice, but for which there is no such evidence for sNfL in the context of MS.^14^ Furthermore in most cases, interpretation was complicated by there being only a single sNfL measurement available, instead of having an available reference within the patient, and the sNfL reference app lacking correction for body mass index (BMI) or kidney function (which was left up for the individual physician to account for).

Our study provides further direction for sNfL implementation and supports further validation using the same questionnaires, in different settings and larger cohorts to ultimately provide robust guidelines for use in daily practice. Based on the described results and experiences in a tertiary MS treatment clinic, in combination with recently published work,^8,21,22^ we recommend using sNfL complementary to standard care with MRI in patients with new MS symptoms: while sNfL was perceived as valuable and indeed impacted clinical decision-making in our center, its temporal relationship with MS disease activity is not yet fully established, and its high specificity with low sensitivity for disease activity should be kept in mind. To improve clinical interpretation, the sNfL result should be compared to a “baseline” reference value in a period of clinical and MRI stability. We do encourage the facilitation of daily NfL measurement in the laboratory, given urgency was within 1 day for almost 20% of cases. Future research should study not only the timing of sNfL in real-world patients with regard to clinical and radiological disease activity but also explore the role, utility, and impact of sNfL in the clinical scenario of disease progression, before full clinical implementation is warranted.

In conclusion, our study shows that sNfL implementation had considerable impact on clinical decision-making and certainty herein, depending on the clinical scenario. Standard implementation of sNfL in clinical practice and regular analysis may very well complement patient management, but warrants caution and further research exploring its exact correct clinical application in different settings.

## Supplemental Material

sj-docx-1-msj-10.1177_13524585241277044 – Supplemental material for Impact of serum neurofilament light on clinical decisions in a tertiary multiple sclerosis clinic Supplemental material, sj-docx-1-msj-10.1177_13524585241277044 for Impact of serum neurofilament light on clinical decisions in a tertiary multiple sclerosis clinic by Zoë YGJ van Lierop, Mark HJ Wessels, Womei ML Lekranty, Bastiaan Moraal, Sam N Hof, Laura Hogenboom, Brigit A de Jong, Nandi Meijs, Liselore A Mensing, Bob W van Oosten, Nik Sol, Zoé LE van Kempen, Lisa Vermunt, Myrthe J Willems, Eva MM Strijbis, Bernard MJ Uitdehaag, Joep Killestein and Charlotte E Teunissen in Multiple Sclerosis Journal

sj-docx-2-msj-10.1177_13524585241277044 – Supplemental material for Impact of serum neurofilament light on clinical decisions in a tertiary multiple sclerosis clinic Supplemental material, sj-docx-2-msj-10.1177_13524585241277044 for Impact of serum neurofilament light on clinical decisions in a tertiary multiple sclerosis clinic by Zoë YGJ van Lierop, Mark HJ Wessels, Womei ML Lekranty, Bastiaan Moraal, Sam N Hof, Laura Hogenboom, Brigit A de Jong, Nandi Meijs, Liselore A Mensing, Bob W van Oosten, Nik Sol, Zoé LE van Kempen, Lisa Vermunt, Myrthe J Willems, Eva MM Strijbis, Bernard MJ Uitdehaag, Joep Killestein and Charlotte E Teunissen in Multiple Sclerosis Journal
