# Supplementary material for: Impact of serum neurofilament light on clinical decisions in a tertiary multiple sclerosis clinic
Source: Mult Scler. 2024 Oct 17;30(13):1620–9. doi: 10.1177/13524585241277044 (PMC11568682; doi:10.1177/13524585241277044)
Supplement: sj-docx-1-msj-10.1177_13524585241277044 – Supplemental material for Impact of serum neurofilament light on clinical decisions in a tertiary multiple sclerosis clinic [file sj-docx-1-msj-10.1177_13524585241277044.docx]

**1. Supplementary material: Questionnaires**

| **Part 1: before disclosure of the sNfL result** | | **Part 2: after disclosure of the sNfL result** |
| --- | --- | --- |
| 1. In which clinical context did you request sNfL? | |  |
|  | □ Differential diagnosis (MS vs. other diagnosis) |  |
|  | □ DMT monitoring |  |
|  | □ New symptoms in a patient with MS |  |
|  | □ DMT baseline |  |
| 2a. If ‘differential diagnosis’ is selected, what do you think is the most likely differential diagnosis (besides multiple sclerosis?) | | 2b. If ‘differential diagnosis’ is selected, with the knowledge of the sNfL result, what do you think is the most likely differential diagnosis (besides multiple sclerosis?) |
|  | □ Leukoaraiosis | □ Leukoaraiosis |
|  | □ Clinically isolated syndrome (CIS) | □ Clinically isolated syndrome (CIS) |
|  | □ Radiologically isolated syndrome (RIS) | □ Radiologically isolated syndrome (RIS) |
|  | □ Neuromyelitis optica (NMO) | □ Neuromyelitis optica (NMO) |
|  | □ Optic neuritis | □ Optic neuritis |
|  | □ Myelopathy | □ Myelopathy |
|  | □ Other neurological diagnosis, namely | □ Other neurological diagnosis, namely |
|  | □ Functional neurological disorder | □ Functional neurological disorder |
|  | □ No neurological diagnosis, namely | □ No neurological diagnosis, namely |
|  | □ No differential diagnosis | □ No differential diagnosis |
| 3a. If 'DMT monitoring' is selected, what is your estimation of DMT efficacy at this moment? | | 3b. If ‘DMT monitoring’ is selected, with the knowledge of the sNfL result, what is your estimation of DMT efficacy? |
|  | □ Effective | □ Effective |
|  | □ Reasonably effective | □ Reasonably effective |
|  | □ Neutral | □ Neutral |
|  | □ Little effective | □ Little effective |
|  | □ Ineffective | □ Ineffective |
| 4a. If 'new symptoms' in a patient with MS is selected, what do you think is the most likely cause of the new symptoms? | | 4b. If ‘new symptoms’ in a patient with MS is selected, with the knowledge of the sNfL result, what do you think is the most likely cause of the new symptoms? |
|  | □ Exacerbation | □ Exacerbation |
|  | □ Pseudo-exacerbation | □ Pseudo-exacerbation |
|  | □ Medication side-effect(s) | □ Medication side-effect(s) |
|  | □ Primary or secondary disease progression | □ Primary or secondary disease progression |
|  | □ I do not know | □ I do not know |
|  | □ Other cause, namely | □ Other cause, namely |
| 5a. How certain do you feel about your answer to the previous question? | | 5b. How certain do you feel about your answer to the previous question? |
|  | □ Certain | □ Certain |
|  | □ Moderately certain | □ Moderately certain |
|  | □ Neutral | □ Neutral |
|  | □ Moderately uncertain | □ Moderately uncertain |
|  | □ Uncertain | □ Uncertain |
| **Part 1: before disclosure of the sNfL result** | | **Part 2: after disclosure of the sNfL result** |
| 6a. Which next step in patient care do you prefer at this moment? | | 6b. With the knowledge of the sNfL result, which next step in patient care do you prefer? |
|  | □ Expectant, return by protocol or in case of symptoms | □ Expectant, return by protocol or in case of symptoms |
|  | □ (Intravenous) methylprednisolone | □ (Intravenous) methylprednisolone |
|  | □ Brain MRI-scan in … weeks | □ Brain MRI-scan in … weeks |
|  | □ Initiate/switch DMT | □ Initiate/switch DMT |
|  | □ Other | □ Other |
| 7. If 'new symptoms' in a patient with MS is selected, what do you want to achieve by obtaining the sNfL result? | |  |
|  | □ Differentiate between MS exacerbation and pseudo-exacerbation |  |
|  | □ Reassurance for the patient |  |
|  | □ In place of other additional investigations |  |
|  | □ Weigh in treatment decision |  |
|  | □ Other |  |
| 8. How motivated are you to obtain the sNfL result? | |  |
|  | □ Highly |  |
|  | □ Moderately |  |
|  | □ Neutral |  |
|  | □ Little |  |
|  | □ Not at all |  |
| 9. Ideally, how urgently do you want to obtain the sNfL result? | |  |
|  | □ Within 2 hours |  |
|  | □ Within 1 day |  |
|  | □ Within 3-4 days |  |
|  | □ Within 7 days |  |
|  | □ Neutral |  |
| 10a. What is your expectation of MS disease activity on the next brain MRI-scan? | | 10b. With the knowledge of the sNfL result, what is your expectation of MS disease activity on the next brain MRI-scan? |
|  | □ Certain | □ Certain |
|  | □ Probable | □ Probable |
|  | □ Neutral | □ Neutral |
|  | □ Unlikely | □ Unlikely |
|  | □ Definitely not | □ Definitely not |
|  |  |  |
|  | | 11. What is your perceived value of the sNfL result in this case on a scale of 1 (low) to 10 (high)? |
