# Supplementary material for: Impact of serum neurofilament light on clinical decisions in a tertiary multiple sclerosis clinic
Source: Mult Scler. 2024 Oct 17;30(13):1620–9. doi: 10.1177/13524585241277044 (PMC11568682; doi:10.1177/13524585241277044)
Supplement: sj-docx-2-msj-10.1177_13524585241277044 – Supplemental material for Impact of serum neurofilament light on clinical decisions in a tertiary multiple sclerosis clinic [file sj-docx-2-msj-10.1177_13524585241277044.docx]

**2. Supplementary material: differential diagnoses**

Overview of answers to the question in the context of ‘differential diagnosis’, what do you think is the most likely differential diagnosis (besides multiple sclerosis?) and the reason for a second opinion at our center (last column).

| Case | Before disclosure  of sNfL | After disclosure  of sNfL | Second opinion about (differentiation between): |
| --- | --- | --- | --- |
| 1 | No other diagnosis | No other diagnosis | RRMS / SPMS |
| 2 | No other diagnosis | No other diagnosis | RRMS and DMT |
| 3 | No other diagnosis | No other diagnosis | PPMS |
| 4 | No other diagnosis | No other diagnosis | PPMS |
| 5 | No other diagnosis | No other diagnosis | SPMS and DMT |
| 6 | No other diagnosis | No other diagnosis | RRMS |
| 7 | No other diagnosis | No other diagnosis | Active RRMS |
| 8 | No other diagnosis | No other diagnosis | Active RRMS |
| 9 | No other diagnosis | No other diagnosis | Leukoaraiosis |
| 10 | No other diagnosis | No other diagnosis | RRMS / PPMS |
| 11 | No other diagnosis | No other diagnosis | Brain tumor |
| 12 | No other diagnosis | No other diagnosis | MOGAD |
| 13 | Functional neurological disorder | Functional neurological disorder | PPMS |
| 14 | Neurosarcoidosis | Neurosarcoidosis | RRMS or both |
| 15 | Mononeuropathy | Mononeuropathy | CNS auto-immunity  (unique oligoclonal bands in CSF) |
| 16 | CNS vasculitis | CNS vasculitis | MS |
| 17 | Brain tumor | Brain tumor | CIS or RRMS |
| 18 | Longitudinal extensive transverse myelitis | Longitudinal extensive transverse myelitis | MOGAD |
| 19 | CRION | CRION | Other opticopathy |
| 20 | Optic neuritis | No other diagnosis | Functional neurological disorder |
| 21 | Optic neuritis | Optic neuritis | CIS |
| 22 | CIS | CIS | Optic neuritis |
| 23 | MOGAD | MOGAD | RRMS |
| 24 | CIS | CIS | MOGAD |
| 25 | Leukoaraiosis | Leukoaraiosis | CIS |
| 26 | Leukoaraiosis | No other diagnosis | Leukoaraiosis |
| 27 | Leukoaraiosis | No other diagnosis | Leukoaraiosis |
| 28 | Leukoaraiosis | Leukoaraiosis | RRMS |
